# Clinical Evidence of Cannabinoids in Migraine: A Narrative Review

**DOI:** 10.3390/jcm11061479

**Published:** 2022-03-08

**Authors:** Flavia Lo Castro, Carlo Baraldi, Lanfranco Pellesi, Simona Guerzoni

**Affiliations:** 1 Department of Biomedical, Metabolic and Neural Sciences, Post-Graduate School in Pharmacology and Clinical Toxicology, University of Modena and Reggio Emilia, 41122 Modena, Italy; locastroflavia@gmail.com; 2 Department of Biomedical, Metabolic and Neural Sciences, PhD School in Neurosciences, University of Modena and Reggio Emilia, 41122 Modena, Italy; infocarlobaraldi@gmail.com; 3 Department of Neurology, Faculty of Health and Medical Sciences, Danish Headache Center, 2100 Copenhagen, Denmark; lanfranco.pellesi@gmail.com; 4 Department of Biomedical, Metabolic and Neural Sciences, Medical Toxicology, Headache and Drug Abuse Research Center, University of Modena and Reggio Emilia, 41124 Modena, Italy

**Keywords:** Δ9-tetrahydrocannabinol, cannabidiol, cannabis, endocannabinoid system, headache, migraine

## Abstract

The endocannabinoid system (ECS) influences many biological functions, and hence, its pharmacological modulation may be useful for several disorders, such as migraine. Preclinical studies have demonstrated that the ECS is involved in the modulation of trigeminal excitability. Additionally, clinical data have suggested that an endocannabinoid deficiency is associated with migraine. Given these data, phytocannabinoids, as well as synthetic cannabinoids, have been tried as migraine treatments. In this narrative review, the current clinical evidence of potential ECS involvement in migraine pathogenesis is summarized. Furthermore, studies exploring the clinical effects of phytocannabinoids and synthetic cannabinoids on migraine patients are reviewed.

## 1. Introduction

Migraine is a primary headache that affects about 16% of the whole population in Western countries, especially middle-aged females [1,2]. Migraine is associated with huge direct and indirect costs, representing one of the most important causes of disability worldwide [3]. The treatment of migraine includes acute and preventive therapies. Despite many options, migraine is still undertreated [4]. Many of these treatments are not very effective or tolerable [5,6]. Thus, the current scenario warrants an exploration of additional options, particularly for patients who do not benefit from or do not tolerate commonly prescribed medications. Medical cannabis is an intriguing alternative to treat migraine. A neuro-modulatory system named the endocannabinoid system (ECS) is formed by endogenous cannabinoids, which are similar in structure and function to compounds of the *Cannabis sativa* plant. More than 60 different cannabinoids are present in *Cannabis sativa*, which has been empirically used to treat headache for a long time [7]. However, there are currently no cannabis-based drugs approved for use in migraineurs. This work briefly discusses the clinical evidence of the pathophysiological role of the ECS in migraine. Moreover, the clinical evidence of the use of *Cannabis sativa* derivatives or similar synthetic compounds in migraine is analyzed.

## 2. Materials and Methods

**Figure 1:**
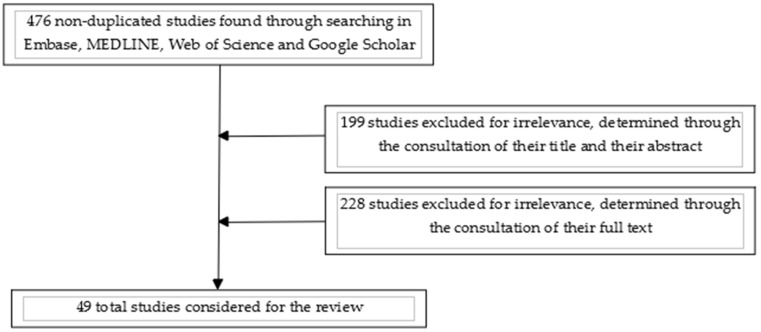
Diagram of the selection process of the cited articles.

A literature review was performed by seraching the following databases: Embase, MEDLINE, Web of Science, Google Scholar and Clinicaltrials.gov, as indicated in a previous article by Bramer et al. [8]. The primary search strategy was conducted using the following MeSH terms: “marijuana/headache” OR “cannabis/headache” OR “marijuana/migraine” OR “cannabis/migraine” OR “endocannabinoids/headache” OR “endocannabinoids/migraine”. Only studies published in the English language before 3 October 2021 were considered. Congress-related published abstracts were not considered. This search retrieved 476 non-duplicated articles, the titles and abstracts of which were subsequently screened for relevance. Among them, 299 were excluded as irrelevant, whilst the rest of the articles were evaluated using full-text analysis. Forty-nine studies were included in this review at the end of the selection process. After these first steps, we decided to proceed with a narrative rather than a systematic review because of the characteristics of the articles found, which are illustrated in detail in the following parts. A simple diagram of the selected publications is presented in Figure 1. 

## 3. Results

### 3.1. Endocannabinoid System and Migraine

The ECS is a neuro-modulatory system that influences many physiological functions, including pain processing and modulation [9]. ECS is composed of: endocannabinoids (eCBs), their receptors (CB) and their synthetic and catabolic enzymes. Arachidonoylethanolamide (AEA) and 2-arachidonoyl-glycerol (2-AG) are the most studied eCBs and act primarily on two isoforms of CB: type 1 (CB1) and type 2 (CB2) [10,11]. CB1 is primarily expressed in neurons, whilst CB2 is mainly expressed in immune cells [12]. It also seems plausible that other eCBs receptors are involved, particularly type 3 CB (CB3), known as GPR55 [13], and the transient receptor potential vanilloid 1 (TRPV1) ion channel [14]. AEA binds both receptor subtypes, with higher selectivity for CB1 than for CB2 [15], whilst 2-AG activates both receptor subtypes as a full agonist [16]. AEA and 2-AG are synthesized from lipid precursors and subsequently released from postsynaptic neurons into the synaptic space [17]. The synthesis of AEA is catalyzed by *N*-acylphosphatidylethanolamine-phospholipase D (NAPE-PLD) [18], whilst that of 2-AG is catalyzed by sn-1-specific diacylglycerol lipase (DAGL) [19]. After their release, eCBs are retrieved via an endocannabinoid membrane transporter (EMT), and AEA is degraded by fatty acid amide hydrolase (FAAH), whereas 2-AG is degraded by monoglyceride lipase (MAGL) [20]. After their release from postsynaptic neurons, eCBs stimulate presynaptic CB1, balancing GABAergic inhibitory activity and glutamatergic excitatory activity. In addition, eCBs may act autocrinally on CB1 and interact with other neurotransmitters, such as dopamine, regulating intrinsic neuronal activity [20]. In 2006, Russo suggested the “clinical endocannabinoid deficiency” (CED), as low levels of eCBs had been reported in painful conditions such as fibromyalgia and migraine [21]. Evidence from several preclinical studies seems to indicate that the dysregulation of ECS, with reduced eCB activity, plays a role in migraine. The key publication by Akerman et al. [22] demonstrated that AEA decreases trigeminovascular system excitability (primarily involved in a migraine attack) in nitroglycerin (NTG)-induced migraine models; in contrast, the most recent preclinical studies have mainly focused on blocking FAAH and MAGL activities, but a complete dissertation of these publications is beyond the scope of this paper (see [23,24,25] for an overview). Clinically, Cupini et al. demonstrated increased activity of FAAH and EMT in platelets in female migraineurs, but not in males [26]. This increased activity, which is not found in tension-type headache sufferers, drove a reduction in the level of AEA and may suggest an imbalance in eCB degradation in women affected by migraine without aura. The same group subsequently observed a significant reduction in FAAH and EMT activity in chronic migraine (CM) and medication overuse headache (MOH) sufferers, the latter being a complication resulting from the frequent use of medicines to treat migraine [27]. However, as the authors had previously observed [26], FAAH and EMT activities were higher in female sufferers of episodic migraine (EM) than in healthy controls and the CM-MOH group. This may be attributable to an adaptive response induced by chronic headache and/or drug overuse [27]. These results were later confirmed by another study, showing that AEA and 2-AG levels were significantly lower in the peripheral platelets of CM sufferers compared to healthy controls [28]. The reduced levels were more evident in females than in males. However, Gouveia-Figueira et al. detected no significant variations in the plasma levels of AEA in EM sufferers [29]. In the cerebrospinal fluid, Sarchielli et al. found that AEA was lower in CM sufferers than healthy controls. In addition, palmitoylethanolamide (PEA) was significantly higher in CM sufferers than in healthy controls, suggesting that higher levels of PEA might represent a compensatory response to the reduced ECS tone in CM [30]. In a positron emission tomography (PET) study, Van der Schueren et al. demonstrated that the binding of a specific CB1 ligand ((18F)MK-9470) to CB1 was augmented in pain-modulating brain areas in the interictal period in female migraineurs compared to controls, suggesting an eCB deficiency [31]. Perrotta et al. found that FAAH activity was significantly reduced after the withdrawal of painkillers, coinciding with clinical improvement. These data were interpreted as indicative of a relationship between AEA levels and the anti-nociceptive effect [32]. Migraine genome-wide association studies did not find specific genetic variants within the ECS [33,34], but Juhasz et al. identified an association between CB1 gene variants and headache with nausea, especially in patients subjected to recent stressful events, indicating the possible role of ECS in patients suffering from life-stress-triggered migraine attacks [35]. Greco et al. found higher CB1 and CB2 levels in mononuclear cells of EM and CM-MOH sufferers compared to healthy controls [36], in accordance with the results on CB1 binding activity in EM [31]. FAAH gene expression was lower in both EM and CM-MOH compared to healthy controls. These data are similar to those reported by Cupini et al. for CM-MOH patients [27] but dissimilar to those reported for EM patients [26,27], even though lower levels of FAAH were detected in CM-MOH in the latter study when compared to the EM group. This indicates a possible dynamic compensatory mechanism to maintain higher AEA levels in a challenged system. NAPE-PLD and DAGL mRNAs were increased in EM and CM-MOH sufferers vs. controls. NAPE-PLD and DAGL mRNA levels were also higher in CM-MOH vs. EM subjects. All of these findings suggest a compensatory mechanism to relieve an eCB deficiency. MAGL mRNAs were also increased in EM and CM-MOH patients: this result, apparently surprising, may be indicative of a higher turnover of 2-AG. Interestingly, all of these changes in the gene expression of different components of the ECS were associated with migraine days. This supports the notion that a dysregulation of the ECS is present in migraine and correlates with the seriousness of migraine. Last year, the plasma levels of AEA and PEA were evaluated in a double-blind, parallel-group clinical study of migraine provocation after receiving sublingual nitroglycerin (0.9 mg). AEA levels increased in both EM patients and healthy controls, whereas PEA increased only in migraine patients, regardless of whether or not a migraine attack was reported. The increased PEA in migraineurs vs. healthy controls presumably reflects migraine-specific mechanisms [37]. No clinical trials on compounds capable of modulating the ECS (such as FAAH/MAGL inhibitors) are currently available for migraine.

### 3.2. Phytocannabinoids

Phytocannabinoids are a group of substances that display a cannabinoid structure and are found in the *Cannabis sativa* plant. To date, about 60 different phytocannabinoids have been described, and their number is still increasing [7], with Δ9-tetrahydrocannabinol (THC) and cannabidiol (CBD) being the most studied ones. Phytocannabinoids have similar chemical formulas but distinct properties that separate them from one another. Despite not having any conclusive scientific evidence, medical cannabis is frequently used by migraine sufferers as a last-resort self-treatment [38]. In a survey-based study conducted in 9003 patients, 121 patients claimed to use cannabis for migraine relief. Interestingly, most of these patients inhaled cannabis, often without informing their general practitioner [39]. In a survey of 145 patients, Aviram et al. found that medical cannabis resulted in a long-term reduction in migraine frequency in >60% of treated patients and was associated with reduced medication intake and less disability [40]. Another study conducted in 589 adult cannabis users reported that migraine sufferers experienced significant migraine relief using medical cannabis [41]. Rhyne et al. retrospectively evaluated the effects of medical cannabis in 121 EM sufferers attending two medical marijuana specialty clinics in Colorado (United States), reporting a global decrease in migraine frequency [42]. However, most of these patients used different formulae of marijuana, even on the same day, and through different routes of administration [42]. Another study explored the effect of different oral formulae of phytocannabinoids in CM sufferers. Patients reported a reduction in pain severity and analgesic consumption after 3 and 6 months of use compared to the baseline, but there were no changes in the number of headache days. The authors concluded that, considering the tonic regulatory role of the ECS, this result may indicate that phytocannabinoids are more useful in pain intensity, rather than frequency [43]. An online survey conducted in 1429 medical cannabis users found that the consumption of phytocannabinoids for migraine treatment often occurred without a physician’s supervision [44]. Despite the reported benefits of cannabis, its therapeutic effects on migraine are influenced by its formulae as well as its route of consumption [45]. Moreover, different cannabinoid formulae may also have different pharmacokinetics, even if taken by the same route [46]. In the above-mentioned study, patients took differently titrated cannabis forms through different routes, thus making it almost impossible to understand which phytocannabinoid is really effective on migraine. The only study that focused on a single route of administration and on three standardized/titrated cannabis forms is the one conducted by Baraldi et al., but the small sample size affected the results [43]. Additionally, the possibility of developing a tolerance exists, since Cuttler et al. found that migraine patients who inhaled medical cannabis used higher doses over time [47]. Another question yet to be answered is if cannabis consumption can lead to the development of MOH. In a preclinical migraine model, the infusion of a cannabinoid receptor agonist, such as THC or WIN55,212-2, seemed to induce latent trigeminal sensitization, thus raising the possibility of MOH development [48]. Moreover, a retrospective study conducted through an electronic chart review found a slight but significant association between MOH and cannabis consumption [49]. No randomized clinical trials investigating phytocannabinoids have been reported in migraine patients. Two trials have been initiated (NCT03972124 and NCT04360044) [50,51], but no results have been published yet. In particular, the first one explores the effect of two dosages of oral cannabidiol vs. placebo in the preventive treatment of CM (NCT03972124) [50], whilst the other one explores the efficacy and safety of inhaled CBD and THC in the acute therapy of migraine attacks, aiming to determine the rate of 2-h pain freedom (NCT04360044) [51].

### 3.3. Synthetic Cannabinoids

Synthetic cannabinoid analogs (SCAs) are non-naturally occurring compounds (e.g., nabilone, HU-210, and dexanabinol) that bind CB1 and CB2. They are fully synthetic and should not be confused with semisynthetic phytocannabinoids (SPs), which are naturally occurring compounds obtained by a partial chemical synthetic process rather than the biosynthetic processes of phytocannabinoids (e.g., CBD converted into dronabinol) [52]. Nabilone is a synthetic cannabinoid CB1 agonist that was previously investigated in a randomized, double-blind, active-controlled crossover study conducted in 30 MOH sufferers [53]. Patients were randomized to receive daily administration of oral nabilone (0.5 mg) or ibuprofen (400 mg). Each treatment period lasted 8 weeks and was performed at home. Nabilone decreased pain intensity and analgesic intake, reduced drug dependence and improved quality-of-life scales. Side effects were not frequent and were mild, and they disappeared after the discontinuation of the therapy. Dronabinol and/or cannabis were successfully utilized in five patients with CM [54]. The efficacy and safety of inhaled dronabinol for the acute treatment of migraine with and without aura were tested in a multicenter, double-blind, placebo-controlled study. The recruitment was completed several years ago, but the results have not yet been published (NCT00123201) [55].

## 4. Discussion

In recent years, migraine researchers have focused mostly on the calcitonin gene-related peptide (CGRP) signaling pathway, leading to the development of novel drugs that target CGRP or its receptor [56]. However, not every migraine patient responds to anti-CGRP medications [57]. In this context, the ECS appears particularly promising as a target for novel drugs. Although phytocannabinoids and synthetic cannabinoids have been associated with important side effects, including dizziness, tachycardia, orthostatic hypotension and psychotic episodes [58], the benefits might exceed the risks. The majority of supporting evidence, however, consists of retrospective studies, online surveys, case series and case reports. Migraine patients enrolled in these studies usually used different cannabis preparations through different routes of administration, making it difficult to explore the therapeutic potential of the Cannabis plant. Proper placebo-controlled trials are needed to establish a therapeutic role for cannabinoids (plant-derived or synthetic) in migraine treatment. Moreover, the results from ECS clinical studies, with very small sample sizes, are not always concordant, possibly reflecting the different methodologies and samples used and the different populations analyzed. A certain limitation of our study is that it is a narrative review and not a systematic one, but considering the aforementioned limits of the studies found (the small sample sizes, the lack of placebo-controlled studies, the often-retrospective design, the different titrations of cannabinoid preparations and the different routes of administration), the results of a systematic review would not have been too dissimilar from a narrative one. The adverse events linked to the modulation of the ECS, increasing eCBs, are still uncertain and should be properly assessed, because, although some authors believe it may be a relatively safe option [59], a recent clinical trial with a FAAH inhibitor (in this case, not used for migraine) was interrupted as a result of serious adverse events [60]. In conclusion, promising data are emerging on the possible role of ECS in migraine. However, the current literature has many gaps, and it has not completely unveiled the real effectiveness and safety of cannabinoids in the treatment of migraine due to the low quality of the studies. Furthermore, it would also be useful to explore the individual therapeutic value of every single cannabinoid in well-designed randomized studies. Moreover, randomized clinical trials are needed to establish the therapeutic role of FAAH/MAGL inhibitors (or other ECS modulators) in migraine. All the studies exploring the use of phytocannabinoids and synthetic cannabinoids in migraine has been summarized in Table 1.

## Figures and Tables

**Table 1:** Clinical studies on phytocannabinoid and synthetic cannabinoid use in migraine.

Reference	Study Design	Number of Patients	Cannabinoid Studied	Route of Administration	Principal Results
Phytocannabinoids					
[39]	Retrospective	9003	Marijuana	Various (inhaled, oral, vaporized, topical)	121 patients used marijuana for migraine self-treatment
[40]	Retrospective, cross-sectional	145	Marijuana	Various (inhaled, oral, vaporized)	>60% patients reported a long-term reduction in migraine frequency
[41]	Retrospective	589	Marijuana	Various (inhaled, oral, vaporized)	86 patients used cannabis for migraine relief; Cannabis was more effective than other painkillers in determining migraine relief
[43]	Retrospective	121	Marijuana	Various (inhaled, oral, vaporized, topical)	Significant decrease in migraine frequency
[43]	Retrospective	32	Bediol^®^, Bedrocan^®^, FM2^®^	Oral	Significant decrease in pain intensity and analgesic consumption after 3 and 6 months of treatment compared to the baseline
[44]	Retrospective	1429	Marijuana	Various (inhaled, oral, vaporized, topical)	35.5% of patients used marijuana to treat their migraines
[45]	Prospective	699	Marijuana	Various (inhaled, oral, vaporized, topical)	94% of patients experienced two-hour migraine relief
[46]	Prospective, crossover	13	Cannabis decoction and cannabis oil	Oral	THC bioavailability is higher for cannabis oil than cannabis decoction
[47]	Prospective	653	Cannabis (both concentrated and flowers)	Inhaled	Self-reported headache and migraine severity were reduced by approximately 50%. Reduction in effectiveness across time
[49]	Retrospective	212	Marijuana	Various (inhaled, oral, vaporized, topical)	Cannabis use significantly decreased migraine frequency
Synthetic cannabinoids					
[51]	Prospective	30	Nabilone 0.5 mg/die	Oral	Nabilone significantly reduced pain intensity and analgesic consumption compared to ibuprofen. Side effects were mild.
[52]	Retrospective	5	Dronabinol 5 mg/die	Oral	Dronabinol significantly reduced migraine frequency compared to the baseline

## Data Availability

Not applicable.
